# Genome-wide association study identifies loci and candidate genes for RVA parameters in wheat (*Triticum aestivum* L.)

**DOI:** 10.3389/fpls.2024.1421924

**Published:** 2024-07-22

**Authors:** Rehmat Ullah, Mingyang Yin, Sen Li, Yasir Israr, Ziyan Wu, Xueping Wang, Jiazheng Yu, Baoyun Li, Zhongfu Ni, Rongqi Liang

**Affiliations:** Frontiers Science Center for Molecular Design Breeding (MOE), Key Laboratory of Crop Heterosis and Utilization (MOE), China Agricultural University, Beijing, China

**Keywords:** common wheat, GWAS, RVA, SNP, starch quality

## Abstract

The gelatinization and retrogradation characteristics of wheat starch affect the eating quality of Chinese-style food. Rapid Visco Analyzer (RVA) parameters have been widely used as important indicators to evaluate and improve the quality of wheat starch. However, the genetic basis of RVA parameters remains to be further explored. In the present study, a natural population was genotyped using 90K single nucleotide polymorphism (SNP) arrays, and the RVA parameters of this population grown in five environments were evaluated. The results showed that 22,068 high-quality SNP markers were identified and distributed unequally on the chromosomes. According to the genetic distance, 214 wheat materials were divided into four groups. Except for the pasting temperature (PTT), six parameters followed a normal distribution. Based on the general linear model, 969 significant association SNPs were detected by genome-wide association studies (GWAS), and chromosomes 7A and 2B had the most associated SNPs. Breakdown viscosity (BV) was associated with the most SNPs (*n* = 238), followed by PTT (*n* = 186), peak viscosity (PV; *n* = 156), trough viscosity (TV; *n* = 127), and final viscosity (FV; *n* = 126). According to the average linkage disequilibrium (LD), 33 stable quantitative trait loci (QTLs) were identified for single parameters in multiple environments, of which 12 were associated with BV, followed by peak time (PT; *n* = 8) and PTT (*n* = 7). On the other hand, 67 pleiotropic QTLs were identified for multiple parameters. Three candidate genes—*TasbeIIa, TasbeI*, and *TassIIa*—were screened for phenotyping analysis. The grain width and the weight of the *TasbeIIa* and *Ta*SS*IIa* knockout (KO) lines were significantly lower than those of the *TasbeI* KO lines and the control (CK). The KO lines had smaller endosperm cells, smaller A-type starch granules, and higher amylose content. The *TasbeI* KO lines showed normal RVA curves, while the *TasbeIIa* KO lines showed flat curves. However, the *TaSSIIa* lines failed to paste under the RVA temperatures. Conclusively, the SNPs/QTLs significantly associated with the RVA parameters and genetic resources with novel haplotypes could be used to improve the quality of wheat starch.

## 1 Introduction

Wheat (*Triticum aestivum* L.) is the second most important food crop in China, accounting for over 20% of the total annual grain production and approximately 25% of the grain crop areas (Regina et al., 2006; Li et al., 2014; Mao et al., 2023). Starch is the main component of wheat grains, accounting for 65%–70% of the dry weight of grains (Rahman et al., 1995). Chinese-style wheat-based food, including noodles, steamed bread, dumplings, and pancakes, are popular not only in China but also across Asia. The starch quality affects the appearance and the eating quality of these types of food and significantly impacts the shelf life and freshness of bread and quick-frozen food items (Miskelly and Moss, 1985; Bao et al., 2002; Goesaert et al., 2005; Zhang et al., 2011; Ma and Baik, 2018). The gelatinization and retrogradation characteristics of starch are important indicators, and the parameters measured using the Rapid Visco Analyzer (RVA) have been widely used in the evaluation of wheat starch quality.

The starch granules in the wheat endosperm could be classified into a large type A and a smaller type B (Meredith, 1981). A-type granules generally form a lens or a disk shape with diameters between 10 and 38 μM, accounting for 70%–80% of the total weight. On the other hand, B-type granules predominantly form a spherical shape with a diameter of less than 10 μM, constituting about 30% of the total weight (Bechtel et al., 1990; Peng et al., 2000; Kim and Huber, 2008). Wheat starch is mainly composed of linear amylose and branched-chain amylopectin, accounting for 20%–25% and 75%–80% of the total starch content in the wheat endosperm, respectively (Graybosch et al., 1998). During the gelatinization of starch, the starch granules absorb water and expand reversibly, the amylose molecules are exuded from the granules, and the starch suspension gradually turns into a viscous paste with high viscosity characteristics. Upon cooling, the paste becomes an elastic gel-like sediment, commonly known as starch retrogradation. The speed of retrogradation mainly depends on the amylose content, as the amylose molecules easily form a regular ordered structure (Crosbie, 1991; Schulman et al., 1994).

There are several enzymes involved in the biosynthesis of starch (Martin and Smith, 1995; James et al., 2003; Tetlow, 2006). For instance, starch synthase (SS), starch-branching enzymes (SBEs), and debranching enzymes regulate the synthesis of amylose and amylopectin within the endosperm. These enzymes are encoded by genes with multiple family members or alleles, which play different roles in the composition and structure of grain starch, thereby affecting the gelatinization and retrogradation characteristics of starch.

The elasticity and the toughness of noodles are mainly influenced by the protein content, the gluten strength, and the rheological properties of the dough. However, the eating quality of noodles, such as the softness, smoothness, and chewiness, is highly correlated with the starch characteristics/flour viscosity. Notably, there exists a significant correlation between the RVA parameters and the noodle quality; hence, these parameters could be used as indicators for the evaluation of starch quality (Crosbie et al., 1992; Araki et al., 1999; Yao et al., 2000; Zhang et al., 2013; Pan et al., 2017). For example, the peak viscosity (PV) of flour was positively correlated with the score of noodles, which could, to some extent, reflect the overall quality of noodles. To achieve a noodle score of 90 or above, the PV value must exceed 270.2 (Liang et al., 2002). Flour with higher viscosity had a greater swelling power and better noodle quality, and the viscosity properties could be used for early generation selection to improve the noodle quality (Konik et al., 1994; Liang et al., 2002; Zhang et al., 2012a; Zhang et al., 2012b; Zhang et al., 2013; Guo et al., 2014; Liu et al., 2018). The starch composition and the viscosity of wheat flour also affect the quality of steamed bread. Steamed bread made from medium-gluten flour with a relatively lower viscosity had proper stickiness, good chewiness, and a high score (Liu et al., 2019; Zhu et al., 2020). In addition, the trough viscosity (TV) of high-quality steamed bread flour ranged from 1,400 to 2,400 cp, while the PV ranged from 2,200 to 3,700 cp (Feng et al., 2016).

A number of quantitative trait loci (QTLs) associated with the RVA parameters have been identified through the construction of mapping populations. Udall et al. (1999) studied the RVA characteristics of starch using a recombinant inbred line (RIL) population and identified nine QTLs located on chromosomes 1AS, 2A, 2B, 2DL, and 3BL. Wu et al. (2008) detected four QTLs linked to PV on chromosomes 1A, 1B, 3A, and 7B and five QTLs related to the breakdown viscosity (BV) on chromosomes 1B, 4A, 5B, 6B, and 7A. In sum, the QTLs of PV and TV were located on 17 chromosomes, while the QTLs for peak time (PT) were located on 10 chromosomes. The QTLs for the final viscosity (FV) were distributed on 19 chromosomes, while the QTLs for setback viscosity (SV) were located on 14 chromosomes (McCartney et al., 2006; Wu et al., 2008; Zhao et al., 2009; Deng et al., 2015; Jin et al., 2016).

In recent years, natural populations have been widely exploited to uncover loci linked with the attributes of wheat quality (Panozzo and Mccormick, 1993). Jin et al. (2016) identified two loci, QFV.caas.5BL.2 and BS0003133_51, that control the FV, which were detected in both environments, explaining 9.9% and 13.6% of the phenotypic variation, respectively. Their genetic distance was 0.01 cM, indicating that the two loci were closely linked. QTLs controlling low TV were detected in both environments, with QTV.caas.1BL and Kukri_C7770_176 accounting for 7.6% and 17.9% of the phenotypic variations of TV, respectively. In addition, marker RAC875_C26860_648 on chromosome 6AS showed highly significant correlations with PV, TV, and BV. These identified loci have important value for future molecular breeding and gene cloning efforts. However, the current genome-wide association studies (GWAS) of wheat starch-related traits based on SNP markers remain limited.

In this study, we collected a set of 214 wheat materials from the major winter wheat planting regions in China and analyzed their genotypes using the Illumina Infinium 90K iSelect platform. Subsequently, a 4-year, five-location field experiment was conducted to evaluate the RVA parameters of wheat flour. The polymorphisms, the SNP markers, and the candidate QTLs for the RVA parameters and their different allelic variations were analyzed in a GWAS. Finally, the functions of three candidate genes related to starch synthesis were investigated. This study provides valuable theoretical insights into the genetic basis of wheat starch quality and offers important genetic resources for the improvement of wheat starch quality through breeding efforts.

## 2 Materials and methods

### 2.1 Plant materials and field trials

A diverse common wheat population of 214 cultivars/lines (**Supplementary Table S1**) mainly from the northern winter wheat region, China, was used in this study.

This winter wheat population was planted in Yangling, Shaanxi Province, from 2014 to 2015 (2015-YL, E1); in Shijiazhuang, Hebei Province, from 2015 to 2016 (2016-SJZ, E2); in Linfen, Shanxi Province, from 2015 to 2016 (2016-LF, E3); in Linfen, Shanxi Province, from 2017 to 2018 (2018-LF, E4); and in Shangzhuang Experimental Station (China Agricultural University), Beijing, from 2017 to 2018 (2018-BJ, E5). Each material at every location had three replicates with a random block design, and each block consisted of four rows with a row spacing of 25 cm and a length of 1.5 m. The field management followed local practices.

### 2.2 SNP genotyping

The genomic DNA from each accession was extracted using the cetyltrimethyl ammonium bromide (CTAB) method. A genome-wide scan of the wheat genotype was performed using Illumina Infinium iSelect 90K SNP markers (Wang et al., 2014). The genotyping of the samples was completed by Boao Jingdian Co. (Beijing, China). The SNP data were analyzed using Genome Studio Polyploid Clustering Module v1.0 software (Illumina Ltd., Hayward, CA, USA).

The obtained genotypes were filtered using the Tassel 5.0 software (Breseghello and Sorrells, 2006), with minimum allele frequency ≥0.05 and more than 10% of missing data. High-quality SNP markers were identified for further analysis.

### 2.3 Population structure and linkage disequilibrium

Polymorphic SNP markers were utilized for genetic clustering analysis using the PowerMarker V3.25 statistical software (Liu and Muse, 2005). The genetic distance between two sets of wheat materials was calculated according to the genetic distance standard of Nei (1972). Subsequently, a phylogenetic tree was constructed using the neighbor-joining (NJ) method and further edited with the MEGA 6 software (Tamura et al., 2013).

The filtered SNP markers were used to calculate the squared allele frequency correlations (*r* ^2^) by pairwise comparisons using the full matrix and sliding window options in Tassel v5.0 (Breseghello and Sorrells, 2006). The linkage disequilibrium (LD) decay distance across the wheat genome was determined as the distance at which *r* ^2^ decreased to half of its maximum value.

### 2.4 RVA parameter measurement

Each clean sample, weighing at least 500 g, was tempered overnight to the 14% moisture content and milled approximately 60% flour extraction using a Brabender Quadrumat Junior Mill (Brabender Inc., Duisberg, Germany). The RVA parameters, including PV, TV, FV, BV, SV, PT, and pasting temperature (PTT), were evaluated with 3.5 g of flour per sample on a 14% moisture basis using a rapid viscosity analyzer (RVA-Super 3, Newport Scientific Co., Warriewood, Australia) according to the AACC (2000) method 76–21.

### 2.5 Genome-wide association study analysis

Correlation analysis between the traits and the markers, principal component analysis (PCA), and kinship analysis were performed using Tassel 5.0 for computational analysis (Breseghello and Sorrells, 2006).

General linear models (GLMs) were used for genome-wide association analysis of the phenotypic traits and genotypic data. Associations between the markers and the parameters were established using the polymorphic SNPs and RVA parameters under different environments and the best linear unbiased prediction (BLUP) values. The significant *p*-value was taken as the threshold (*p* = 0.001) in the wheat SNP whole-genome association analysis. Statistical analysis showed that the *p*-value was ≤0.001, indicating a highly significant association between the locus and the parameter.

### 2.6 Analysis of putative candidate genes

To determine the potential candidate genes, the NCBI database and the reference genome annotations from IWGSC v2.0 were used to search for high-confidence genes that were close to the identified SNPs by examining the LD decay interval of the peak markers, typically around 4.0 Mb.

Three single guide RNA (sgRNA) target sequences of the candidate genes were designed using the E-CRISPR design website (http://www.e-crisp.org/E-CRISP/designcrispr.html). After amplification with two pairs of primers (**Supplementary Table S2**), the sgRNAs were cloned into the pBUE411 vector. The CRISPR–Cas9 constructs were transformed into the wheat cultivar Fielder using *Agrobacterium tumefaciens*-mediated (strain EHA105) transformation (Ishida et al., 2015). The seeds from knockout (KO) plants were planted using the pedigree method. Each KO plant in every generation was screened through PCR and sequencing until homozygous KO lines were obtained. The phenotypes and RVA parameters were analyzed for the KO lines grown in the isolated field of Shangzhuang Experimental Station (China Agricultural University), Beijing. The cross-sections of the dry mature grains were coated with gold and then observed using a Hitachi S-3400N (Hitachi, Tokyo, Japan) scanning electron microscope (Liu et al., 2023).

## 3 Results

### 3.1 Genotyping and population structure

A total of 22,068 high-quality SNP markers were identified, which were distributed on all 21 chromosomes, of which the SNP markers 8375, 11089, and 2604 were located in the A, B, and D subgenomes, respectively (**Supplementary Figure S1A**). The distribution of the SNP markers on each chromosome is illustrated in **Supplementary Figure S1B**. The decay patterns of LD varied depending on the A, B and D subgenomes. The average LD decay distance for the whole genome was approximately 4.0 Mb (**Supplementary Figure S2**).

**Figure 1:**
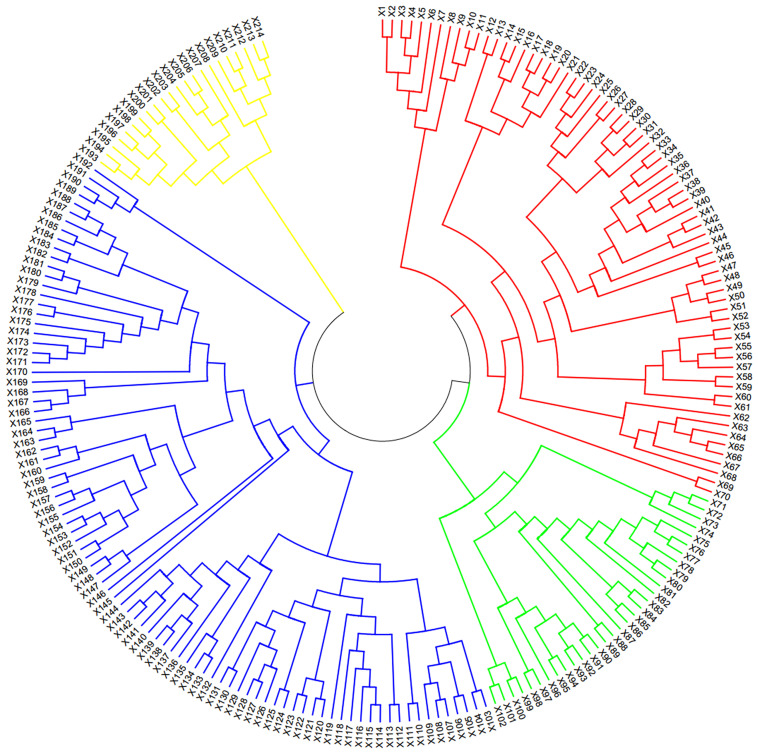
Neighbor-joining tree of 214 wheat materials.

The NJ phylogenetic tree consisted of four groups based on the SNP genetic distance (**Figure 1**). The GI group, represented by the red portion in the clustering map, contained 70 wheat cultivars (lines), predominantly cultivated in Henan Province. The GII group, depicted in the green portion, included 32 cultivars (lines), mainly cultivated in Shandong Province. The GIII group, indicated by the blue part, encompassed 90 cultivars (lines) and was further divided into four subgroups. These subgroups mainly originated from Hebei Province and underwent recurrent selection using the French cultivar ‘Renan’ (III-1), using the Spelt ancestry (III-2), and using ear-branched wheat (III-3), as well as a mix of cultivars from Henan and Shandong Province (III-4). The GIV group, the yellow part in the figure, included 22 materials from different sources. Conclusively, the genetic structures of these materials met the requirements for GWAS analysis.

### 3.2 Phenotypic evaluation

In order to analyze the phenotypic variations in the RVA parameters within this natural population, samples from five different environments were examined. The results (**Table 1**) revealed that the population exhibited high genetic variations in the RVA parameters, with the average broad-sense heritabilities exceeding 50%, indicating that these phenotypic variations mainly resulted from the different genetic backgrounds. According to the single-sample Kolmogorov–Smirnov test, six parameters (except PTT) followed a normal distribution (**Supplementary Figure S3**; **Table 1**), indicating that these RVA parameters were controlled by multiple genes.

**Table 1:** Phenotypic data and broad-sense heritabilities (*h* ^2^) of seven Rapid Visco Analyzer (RVA) parameters under five environments and best linear unbiased prediction (BLUP) values.

Trait	Environment	Max. value	Min. Value	Mean	SD	CV (%)	*h* ^2^ (%)	Kurtosis	Skewness
PV	E1	322.58	98.79	232.13	37.65	16.22	64.10	1.13	−0.74
	E2	363.88	125.83	240.60	40.00	16.63	70.35	0.19	0.10
	E3	320.79	118.63	217.78	34.88	16.02	67.54	0.35	−0.15
	E4	353.50	199.69	273.88	26.36	9.62	74.69	0.11	−0.21
	E5	350.83	87.79	207.60	49.23	23.71	63.56	−0.10	−0.26
	BLUP	342.32	126.15	234.40	37.62	16.44	68.05	0.34	−0.25
TV	E1	202.67	27.38	149.45	27.94	18.70	67.30	2.86	−1.30
	E2	222.67	43.25	154.04	30.33	19.69	71.47	1.14	−0.69
	E3	197.33	37.96	137.33	28.04	20.42	67.81	0.97	−0.81
	E4	232.67	125.53	192.59	19.39	10.07	66.37	0.87	−0.80
	E5	229.69	25.22	127.13	43.40	34.14	63.73	−0.48	−0.24
	BLUP	217.01	51.87	152.11	29.82	20.60	67.34	1.07	−0.77
BV	E1	155.67	28.81	85.72	15.80	18.43	76.44	2.00	0.25
	E2	150.63	35.67	86.50	17.42	20.14	71.77	0.62	0.37
	E3	149.25	26.25	81.42	15.40	18.91	74.19	1.56	0.24
	E4	146.36	33.38	81.28	14.91	18.34	86.60	1.86	0.29
	E5	139.08	32.75	80.48	13.96	17.35	71.21	1.59	0.10
	BLUP	148.20	31.37	83.08	15.50	18.63	76.04	1.53	0.25
FV	E1	340.33	68.58	270.29	42.91	15.88	65.41	3.27	−1.31
	E2	352.39	125.53	264.56	40.93	15.47	67.89	0.89	−0.57
	E3	343.92	94.17	247.27	44.27	17.90	65.09	0.73	−0.74
	E4	364.39	217.56	311.85	24.28	7.79	60.87	2.22	−0.11
	E5	333.94	54.78	207.38	59.76	28.82	62.46	−0.37	−0.40
	BLUP	346.99	112.12	260.27	42.43	17.17	64.34	1.35	−0.63
SV	E1	156.28	41.21	121.05	16.34	13.50	69.13	3.42	−1.15
	E2	141.17	66.08	110.94	12.60	11.36	48.46	0.52	−0.34
	E3	146.58	37.63	110.33	18.49	16.76	63.75	1.81	−1.04
	E4	137.81	90.81	119.26	8.55	7.17	52.39	0.70	−0.64
	E5	115.86	29.56	80.42	18.00	22.38	49.33	−0.03	−0.52
	BLUP	139.54	53.06	108.4	14.80	14.23	56.61	1.28	−0.74
PT	E1	6.38	5.20	6.03	0.20	3.32	58.00	3.35	−1.55
	E2	6.51	5.33	6.17	0.19	3.08	71.34	3.77	−1.67
	E3	6.33	4.76	5.96	0.25	4.19	59.77	4.45	−1.77
	E4	6.60	5.87	6.34	0.14	2.21	63.91	0.48	−0.73
	E5	6.56	4.84	5.97	0.33	5.53	62.08	0.93	−0.96
	BLUP	6.48	5.2	6.09	0.222	3.67	63.02	2.60	−1.34
PTT	E1	86.45	71.05	83.49	1.81	2.17	34.08	14.60	−3.06
	E2	87.25	66.18	83.33	3.74	4.49	64.86	8.55	−2.93
	E3	86.67	76.17	84.00	1.54	1.83	42.53	4.10	−1.53
	E4	85.85	65.25	82.54	3.96	4.80	48.04	7.88	−2.77
	E5	86.05	71.52	82.17	2.51	3.05	64.96	3.55	−1.54
	BLUP	86.45	70.03	83.11	2.71	3.27	50.89	7.74	−2.37

SD, standard deviation; CV, coefficient of variation; PV, peak viscosity; TV, trough viscosity; BV, breakdown viscosity; FV, final viscosity; SV, setback viscosity; PT, peak time; PTT, pasting temperature; E1, 2015-YL; E2, 2016-SJZ; E3, 2016-LF; E4, 2018-LF; E5, 2019-BJ

Based on the 3-year phenotype data from five locations, 20 materials with extreme phenotypic values were selected for genetic breeding (**Supplementary Table S3**). The cultivar X110 (Xinong 94) exhibited the lowest PV, TV, SV, and FV values, while the line X159 (Fen-1) had the highest PV, TV, and FV values among the natural population.

**Figure 2:**
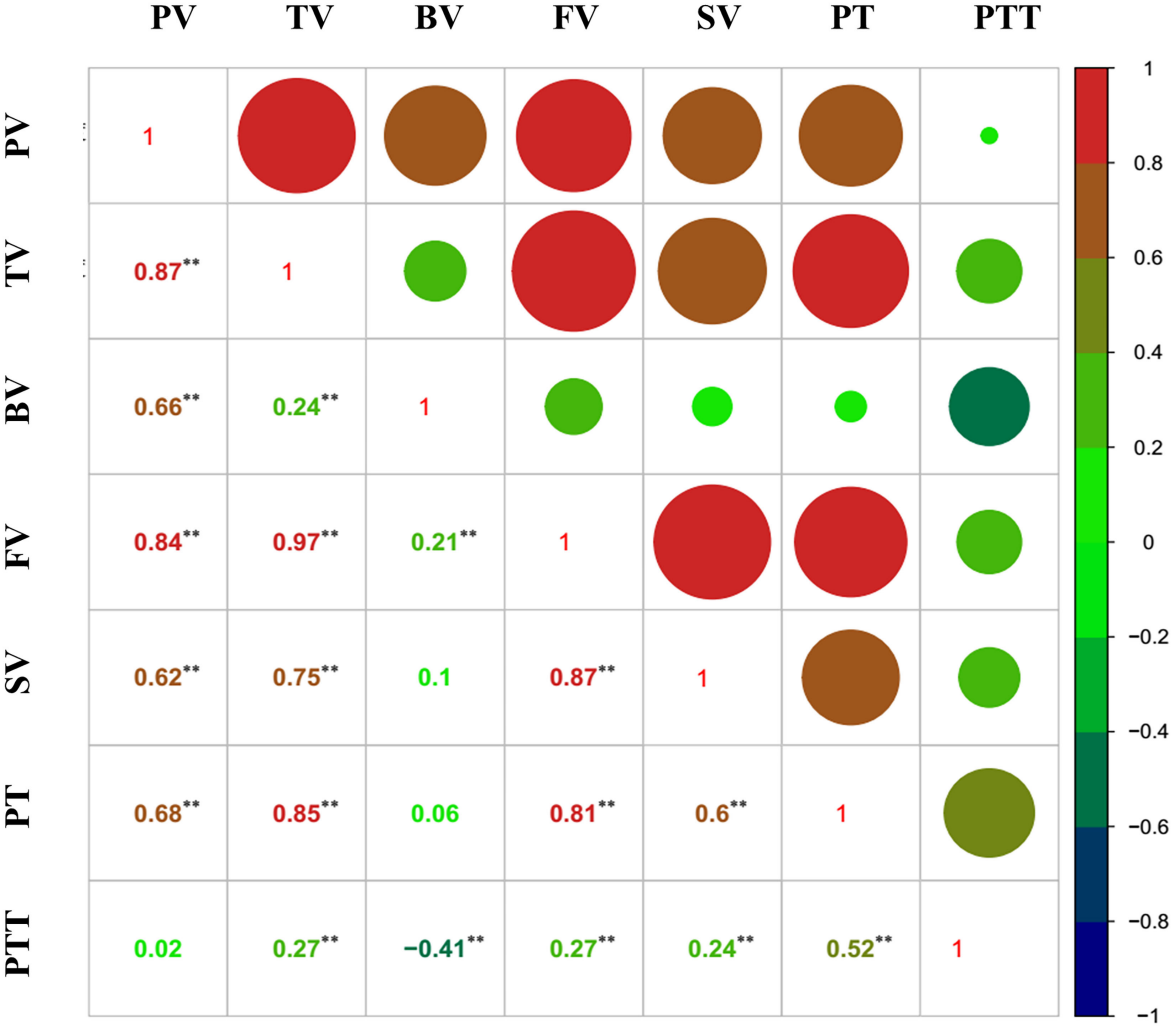
Correlation analysis of the Rapid Visco Analyzer (RVA) parameters. **, p < 0.01.

Except for three weak correlations, the other correlations between the RVA parameters reached significance (*p* < 0.05) (**Figure 2**), indicating that these parameters may be simultaneously associated with some of the SNP markers/QTLs.

### 3.3 GWAS of the RVA parameters under five environments

GWAS analysis of the seven RVA parameters in five environments showed that a total of 969 SNPs (*p* < 0.001) were significantly associated with these parameters, with the explanation rates of the phenotypic variation for a single locus ranging from 5.60% to 30.56%. Of these, 269 SNPs were significantly correlated (*p* < 0.0001) and were mainly distributed on chromosomes 7A (*n* = 87), 2D (*n* = 42), 2A (*n* = 31), and 2B (*n* = 27).

The SNP markers were detected in 20 chromosomes, except for Chr4D (**Supplementary Figure S4**). Chr7A had the highest number of associated SNPs (*n* = 211), with each SNP explaining 5.66%–19.30% of the phenotypic variation. Chr2B had the second highest number of SNPs (*n* = 140), and each explained 5.61%–18.34% of the phenotypic variation. The uneven distribution of SNPs across different chromosomes indicated that their identification was affected by marker density and the RVA parameters.

#### 3.3.1 SNP markers associated with the RVA parameters

Among the seven RVA parameters, BV was associated with the most SNPs (*n* = 238), accounting for 24.56%, followed by PTT (*n* = 186), PV (*n* = 156), TV (*n* = 127), FV (*n* = 126), SV (*n* = 78), and PT (*n* = 58).

The significant (*p* < 0.001) SNP markers associated with BV were mainly distributed on chromosomes 7A, 2D, and 6A, and each explained 5.73%–13.66% of the phenotypic variation. Among them, 94 SNPs were highly significant (*p* < 0.0001). Excalibur_c49801_345 on Chr1D (476.26 Mb) was detected in 2015-YL, 2016-LF, and 2018-LF, explaining 10.51%, 13.66%, and 10.73% of the variation, respectively.

The significant (*p* < 0.001) SNPs associated with PTT were mainly distributed across chromosomes 7A and 2D. Of these, 34 were highly significant loci (*p* < 0.0001), and each accounted for 5.80%–18.21% of the phenotypic variation. Tdurum_contig27843_375 on Chr1B (421.67 Mb) had the highest rate of contribution and was only detected in 2015-YL.

The significant (*p* < 0.001) SNPs associated with TV were primarily distributed on chromosomes 2B (2016-LF) and 6B (2018-BJ), and each explained 6.13%–15.2% of the phenotypic variation. Among them, 16 SNPs reached a highly significant level (*p* < 0.0001). Kukri_c33620_129 on Chr7A (717.26 Mb), with a contribution rate of 15.2%, was detected in 2015-YL.

The significant (*p* < 0.001) SNPs associated with FV were mainly distributed on chromosomes 2B (2016-LF), 6B (2018-BJ), and 1A (2016-LF), and each explained 5.52%–13.71% of the phenotypic variation. BS00028092_51 on Chr2B (173.94 Mb) was highly significant (*p* < 0.0001) and was detected in 2016-SJZ, explaining 13.71% of the variation.

The SNPs (*p* < 0.001) associated with PV were mainly located on chromosomes 7A (2105-LF, 2016-SJZ, and 2016-LF), 6A (2018-LF), 6B (2018-BJ), and 1A (2015-YL). Among them, five SNPs had a highly significant correlation (*p* < 0.0001), and each explained 5.95%–12.18% of the phenotypic variation. Kukri_C33620_129 on Chr7A (717.26 Mb) and BS00110297_51 on Chr7A (207.82 Mb) explained 12.18% and 11.91% of the phenotypic variation, respectively.

The significant (*p* < 0.001) SNPs associated with SV were mainly distributed on chromosomes 2B (2013-LF) and 5A, and their explanation rates for the phenotypic variation ranged from 5.61% to 19.30%. Of these SNPs, 21 reached a highly significant level (*p* < 0.0001). Kukri_c33620_129 on Chr7A (717.26 Mb), which was detected in 2015-YL, had the highest explanation rate of 19.30%.

The significant (*p* < 0.001) SNPs associated with PT were mainly distributed on chromosomes 6B, 2B, and 2A. Among them, 10 highly significant (*p* < 0.0001) SNPs explained 5.60%–18.97% of the phenotypic variation, with RAC875_c93959_96 on Chr 6A (594.96 Mb) having the highest explanation rate, accounting for 18.97% of the variation in 2016-SJZ.

#### 3.3.2 Stable QTLs of the single RVA parameters

The stable significant SNPs (detected in at least two environments) within a 4.0-Mb region were combined as stable QTLs according to the average LD in the genomes of the population; thus, 33 stable QTLs were identified for the RVA parameters (**Table 2**).

**Table 2:** Stable quantitative trait loci (QTLs) identified simultaneously in at least two environments.

Trait	QTL	No. of SNPs	Chr	Pos. (Mb)	Environment	*R* ^2^ min. (%)	*R* ^2^ max. (%)
BV	*qBV1B*	1	1B	421.67	E1, E3, E4, E5	9.61	12.01
	*qBV1D*	1	1D	476.26	E2, E3, E4	5.95	8.21
	*qBV2B*	1	2B	789.44	E1, E3, E4, E5	9.57	13.66
	*qBV2D*	16	2D	641.96–650.06	E1, E2, E3, E4, E5	6.45	12.63
	*qBV3B*	1	3B	733.82	E1, E3, E4, E5	10.13	11.74
	*qBV5B*	1	5B	513.59	E1, E3, E4, E5	6.48	16.85
	*qBV6A*	1	6A	420.09	E4, E5	6.60	11.58
	*qBV6B*	1	6B	59.01	E1, E3, E4, E5	9.61	12.01
	*qBV7A.1*	12	7A	232.38–240.75	E1, E2, E3	6.32	10.36
	*qBV7A.2*	2	7A	659.37	E1, E2, E3, E4, E5	6.46	11.41
	*qBV7D*	2	7D	220.29–224.47	E1, E2, E3	7.01	8.63
PT	*qPT2A.1*	1	2A	674.15	E1, E2, E4	9.48	10.12
	*qPT2A.2*	2	2A	728.78	E2, E3/E4	8.81	13.73
	*qPT2A.3*	2	2A	763.69–765.71	E1, E4	6.10	7.00
	*qPT2A.4*	1	2A	801.22	E1, E4	6.15	6.38
	*qPT2B*	1	2B	782.53	E2, E4	12.60	15.37
	*qPT2D*	1	2D	648.21	E1, E4	9.66	12.22
	*qPT3A*	1	3A	646.85	E1, E2, E4	10.28	12.41
	*qPT5A*	1	5A	706.24	E1, E3	7.98	7.87
	*qPT7A*	1	7A	4.01	E2, E3	7.90	11.25
PTT	*qPTT2A*	1	2A	728.78	E2, E4	13.59	13.73
	*qPTT2D*	1	2D	648.21	E1, E4	9.66	12.22
PV	*qPV7A*	11	7A	232.59–240.75	E1, E2, E3,E4	5.95	7.80
	*qPV7D*	2	7D	220.29–224.47	E1, E2, E3	5.96	6.59
SV	*qSV2B*	1	2B	17.39	E2, E3	12.73	15.31
TV	*qTV2A*	1	2A	728.78	E2, E3	8.62	8.62
	*qTV2B.1*	1	2B	157.70	E2, E3	6.13	6.73
	*qTV2B.2*	1	2B	165.51	E1, E3	6.32	6.59

*R* ^2^ is the phenotypic variance explained.

Chr, chromosome; Pos., position; BV, breakdown viscosity; PT, peak time; PTT, pasting temperature; PV, peak viscosity; SV, setback viscosity; TV, trough viscosity.

There were 39 SNPs associated with BV detected stably in two or more environments: three were detected in all environments, six in four environments, and 21 in three environments. These SNPs were predominantly distributed on chromosomes 7A and 2D and were combined with 11 QTLs. The QTLs qBV2D and qBV7A.2 were identified in all environments and explained more than 11% of the variation.

There were nine stable QTLs controlling PT. The QTL qPT2A, which was linked to Ku_c40588_1376 on Chr2A (728.78 Mb), was detected in 2016-LF (*p* < 0.001) and 2016-SJZ (*p* < 0.0001), explaining 8.81% and 13.57% of the phenotypic variation, respectively.

There were two stable QTLs controlling PTT. The stable QTL qPTT2A, which was linked to Ku_c40588_1376 on Chr2A (728.78 Mb), was detected in 2016-SJZ and 2018-LF, while qPTT2D, linked to BobWhite_c1971_839 on Chr2D (648.21 Mb), was detected in 2018-LF and 2015-YL.

A total of 11 SNPs associated with PV were detected in four environments, explaining 5.95%–7.80% of the variation, and were combined with the QTL qPV7A located on Chr7A (232.59–240.75 Mb). Another stable QTL, qPV7D on Chr7D (220.39–224.47 Mb), contributed 5.96%–6.59% of the PV variation.

The stable QTL qSV2B that controlled SV, which was linked to BS00028092_51 on Chr2B (13.79 Mb), was detected in 2016-LF and 2016-SJZ, with explanation rates of 12.73% and 15.31%, respectively.

Three stable QTLs controlling TV were detected in multiple environments. The QTL qTV2B.1 linked to JD_c767_567 was detected in 2016-LF and 2016-SJZ, explaining 6.73% and 6.13% of the variation, respectively. The QTL qTV2B.2 linked to Kukri_c34353_821 was stably detected both in 2016-LF and 2015-YL, contributing 6.59% and 6.32% of the phenotypic variation, respectively. The stable QTL qTV2A, linked to Ku_c40588_1376 on Chr2A (728.78 Mb), was detected in 2016-SJZ and 2016-LF.

#### 3.3.3 Pleiotropic loci of two or more parameters

A total of 162 polymorphic SNPs associated with two or more parameters were mainly distributed on chromosomes 1B, 2A, 2B, 2D, 6A, 6B, and 7A, and the explanation rates of the phenotypic variation for a single locus ranged from 5.61% to 30.56% (**Supplementary Table S4**). Based on the average LD in the genomes of the population, 67 QTLs were thus identified for two or more RVA parameters (**Table 3**).

**Table 3:** Pleiotropic quantitative trait loci (QTLs) identified simultaneously with two or more Rapid Visco Analyzer (RVA) parameters.

QTL	No. of SNPs	Chr.	Pos. (Mb)	RVA parameter (environment)	*R* ^2^, min–max (%)
*qRVA1A.1*	5	1A	301.63–306.98	PV(E1), FV(E3)	8.32–9.25, 6.08–6.70
*qRVA1B.1*	1	1B	16.22	PV(E5), TV(E5)	9.97, 8.75
*qRVA1B.2*	1	1B	421.67	BV(E1, E3,E4,E5), PTT(E1)	10.56–13.50, 18.21
*qRVA1B.3*	2	1B	566.99	TV(E2), SV(E2)	6.65, 5.94
*qRVA1B.4*	3	1B	626.71	TV(E1), FV(E1)	6.39–7.00, 8.11–8.55
*qRVA1B.5*	1	1B	638.93	PT(E3), PTT(E3)	6.24, 6.26
*qRVA1D.1*	1	1D	457.15	TV(E1), FV(E1)	6.57, 8.21
*qRVA1D.2*	1	1D	476.26	PV(E4), BV(E1,E3,E4), PTT(E1)	9.41, 10.51–13.66, 18.77
*qRVA2A.1*	1	2A	15.88	FV(E2), PT(E2), PTT(E1)	7.61, 11.79, 8.98
*qRVA2A.2*	1	2A	40.82	FV(E1), PT(E1)	8.11, 9.87
*qRVA2A.3*	2	2A	700.96–703.55	TV(E1, E2), FV(E1), SV(E1,E2), PT(E1)	7.15–10.58, 10.07, 7.79–8.62, 10.72
*qRVA2A.4*	2	2A	728.78–734.26	PV(E2), TV(E2,E3), FV(E1,E2), SV(E1), PT(E2,E3), PTT(E2,E3,E4)	11.3, 8.62, 6.95–10.07, 8.14, 8.81–13.05, 13.59–13.94
*qRVA2A.5*	2	2A	743.10	TV(E1), FV(E1)	6.38, 6.95
*qRVA2B.1*	2	2B	12.88–17.39	FV(E2), SV(E2,E3), PT(E3), PTT(E3)	13.71, 12.73–15.31, 8.98, 7.03
*qRVA2B.2*	2	2B	131.57	PV(E3), FV(E3), SV(E3)	6.57, 6.56–7.35, 5.84–6.64
*qRVA2B.3*	3	2B	140.78–141.56	FV(E3), SV(E3)	6.56–6.60, 5.84–5.89
*qRVA2B.4*	13	2B	154.99–160.20	TV(E2,E3), FV(E3), PT(E3),	6.13–11.26, 6.51–9.15, 5.88–5.96
*qRVA2B.5*	1	2B	165.51	PV(E1), TV(E1,E3), FV(E1), PT(E1)	6.50, 6.59–6.32, 6.32, 8.74
*qRVA2B.6*	1	2B	249.53	PV(E1), FV(E3)	6.15, 6.04
*qRVA2B.7*	1	2B	326.76	PV(E1), TV(E3), FV(E3)	10.11, 13.32, 8.16
*qRVA2B.8*	1	2B	404.43	TV(E3), FV(E3), SV(E3)	8.84, 7.91, 7.46
*qRVA2B.9*	2	2B	427.95–429.14	FV(E3), SV(E3)	5.92–7.91, 5.61–6.70
*qRVA2B.10*	1	2B	658.61	PV(E3), TV(E3), FV(E3)	6.35, 8.23, 6.43
*qRVA2B.11*	1	2B	768.55	TV(E4), FV(E4), PT(E4)	8.88, 10.96, 11.43
*qRVA2B.12*	3	2B	782.53–789.44	PV(E3), BV(E1,E3,E4,E5), FV(E3), SV(E3), PT(E2), PTT(E1,E2,E4)	6.69, 9.45–12.01, 8.34–8.89, 12.40, 12.60–16.68
*qRVA2D.1*	1	2D	636.07	FV(E3), SV(E3)	10.20, 8.67
*qRVA2D.2*	15	2D	641.96–650.32	PV(E3,E5), BV(E1,E2,E3,E4,E5), PTT(E1,E4)	8.48–9.67, 6.48–12.63, 9.19–17.07
*qRVA3A.1*	2	3A	686.78–686.79	FV(E3), SV(E3)	6.01–6.02, 6.79–6.81
*qRVA3A.2*	1	3A	714.95	PT(E3), PTT(E3)	5.95, 6.02
*qRVA3B.1*	1	3B	733.82	BV(E1,E3,E4,E5), PTT(E1)	10.13–11.74, 17.16
*qRVA4A.1*	1	4A	596.77	PT(E3), PTT(E3)	5.95, 5.81
*qRVA4A.2*	1	4A	688.10	BV(E4), PTT(E1)	7.59, 13.91
*qRVA4A.3*	1	4A	741.99	FV(E2), PT(E2)	6.90, 10.82
*qRVA4D.1*	2	4D	121.18	TV(E2), PT(E3), PTT(E3)	6.17, 9.03–10.75, 7.91–8.27
*qRVA5A.1*	1	5A	362.78	TV(E1), FV(E1), SV(E1)	8.63, 7.30, 6.10
*qRVA5A.2*	1	5A	414.05	TV(E2), FV(E2), SV(E2), PT(E2)	7.96, 8.67, 7.91, 11.88
*qRVA5A.3*	1	5A	570.19	FV(E3), SV(E3)	6.60, 6.72
*qRVA5B.1*	2	5B	10.52–11.46	TV(E1), FV(E1), SV(E1)	10.95, 6.41–10.14, 6.54–7.73
*qRVA5B.2*	1	5B	513.59	PV(E5), BV(E1,E4,E5), PTT(E1)	8.74, 9.16–11.53, 16.57
*qRVA5D.1*	1	5D	3.61	PV(E2), TV(E2), FV(E2), SV(E2)	9.13, 7.88, 9.31, 11.81
*qRVA5D.2*	1	5D	31.27	PV(E2), BV(E2)	6.25, 6.86
*qRVA5D.3*	1	5D	494.81	TV(E1), FV(E1), SV(E1), PT(E1)	7.00, 9.24, 10.05, 7.14
*qRVA5D.4*	1	5D	539.22	TV(E2), PT(E2)	7.18, 6.68
*qRVA6A.1*	1	6A	9.32	TV(E1), PT(E1)	8.01, 9.11
*qRVA6A.2*	2	6A	402.95–410.92	PV(E4), BV(E4)	6.31–6.36, 6.30–6.37
*qRVA6A.3*	2	6A	420.09–424.09	PV(E4), BV(E4,E5)	6.35–6.36, 6.30–6.85
*qRVA6A.4*	10	6A	430.93–440.06	PV(E4), BV(E4)	6.27–6.57, 6.30–6.55
*qRVA6A.5*	1	6A	594.96	FV(E2), PT(E2)	10.91, 18.97
*qRVA6B.1*	1	6B	59.01	BV(E1,E3,E4,E5), PTT(E1)	9.61–12.01, 16.69
*qRVA6B.2*	7	6B	664.38–668.43	PV(E5), TV(E5), FV(E5), PT(E5)	6.94–7.42, 7.27–8.90, 6.85–8.36, 6.78–7.45
*qRVA6D.1*	1	6D	441.91	PV(E5), TV(E5)	6.99, 6.86
*qRVA6D.2*	2	6D	464.94–465.96	FV(E2), SV(E2)	7.39–7.45, 8.54–8.78
*qRVA7A.1*	2	7A	17.34–20.78	PV(E3), TV(E1), FV(E1,E3), SV(E1)	11.91, 10.94, 10.01–11.07, 9.47
*qRVA7A.2*	1	7A	35.60	TV(E4), FV(E4)	8.05, 8.62
*qRVA7A.3*	1	7A	64.73	PV(E3), BV(E3)	7.07, 6.85
*qRVA7A.4*	13	7A	232.38–240.75	PV(E1,E2,E3), BV(E1,E2,E3)	5.95–7.80, 6.34–10.36
*qRVA7A.5*	1	7A	244.47	PV(E3), BV(E3)	6.99, 8.19
*qRVA7A.6*	13	7A	263.73–273.46	BV(E3), PTT(E3)	5.73–6.32, 6.22–7.11
*qRVA7A.7*	1	7A	277.73	BV(E3), PTT(E2)	6.59, 6.29
*qRVA7A.8*	5	7A	281.37–285.17	TV(E3), BV(E3), PTT(E2,E3)	6.32, 6.32–7.17, 6.21–6.25
*qRVA7A.9*	2	7A	659.37	BV(E1,E2,E3,E4,E5), PTT(E1)	6.46–11.41, 16.62
*qRVA7A.10*	1	7A	717.26	PV(E1), TV(E1), SV(E1), PT(E1), PTT(E1)	12.18, 15.20, 19.30, 12.21, 13.89
*qRVA7B.1*	1	7B	348.95	BV(E3), PTT(E2)	6.77, 6.29
*qRVA7B.2*	1	7B	743.74	TV(E4), FV(E4)	10.50, 7.86
*qRVA7D.1*	2	7D	220.29–224.47	PV(E1,E2,E3), BV(E1,E2,E3)	5.96–6.59, 7.01–8.63
*qRVA7D.2*	1	7D	252.55	BV(E3), PTT(E2)	5.73, 6.25
*qRVA7D.3*	1	7D	518.78	PV(E5), TV(E5)	7.14, 6.75

*R* ^2^ is the phenotypic variance explained.

Chr, chromosome; Pos., position; PV, peak viscosity; TV, trough viscosity: BV, breakdown viscosity; FV, final viscosity; SV, setback viscosity; PT, peak time; PTT, pasting temperature.

Remarkably, three QTLs exhibited significant associations with five or six parameters simultaneously. The QTL *qRVA2A.4* was stably detected in 2016-LF, 2018-LF, and 2016-SJZ and was significantly associated with PTT, PT, PV, FV, SV, and TV, with a contribution rate of 6.95%–13.94% of the respective variation. The QTL *qRVA2B.12* was associated with PV, BV, FV, SV, PT, and PTT, explaining 6.69%–16.68% of the corresponding variation. *qRVA7A.10* had a highly significant effect on PV, PT, PTT, SV, and TV in 2016-YL, explaining 12.18%–19.30% of the corresponding variation, but was not detected in other environments.

Eight QTLs controlling four parameters simultaneously were detected and distributed on chromosomes 5D, 2B, 2A, 5A, 6B, and 7A, with contribution rates ranging from 6.32% to 12.73%. Three QTLs—*qRVA2A.3*, *qRVA2B.1*, and *QRVA7A.1*—were significantly associated with SV, FV, and two other parameters and were detected in two environments. The remaining five QTLs were detected in only one environment.

There were 14 QTLs simultaneously controlling three parameters that were detected and distributed on Chr2B, followed by chromosomes 5B, 1A, 1D, 2D, 4D, 5A, and 7A, with the contribution rate of each locus ranging from 5.88% to 18.17%. Notably, *qRVA1D.2*, *qRVA2D.2*, and *qRVA5B.2* simultaneously controlled PV, BV, and PTT and were detected in three to five environments.

There were 42 QTLs controlling two parameters simultaneously, with the most QTLs on Chr7A (*n* = 7), followed by Chr1B (*n* = 5) and Chr6A (*n* = 5). Four QTLs—*qRVA1B.2*, *qRVA3B.1*, *qRVA6B.1*, and *qRVA7A.9*—controlling BV and PTT simultaneously were detected in four or five environments. Two QTLs, *qRVA7A.4* and *qRVA7D.1*, controlling BV and PV simultaneously were detected in three environments.

### 3.4 Phenotype verification of significant loci

**Figure 3:**
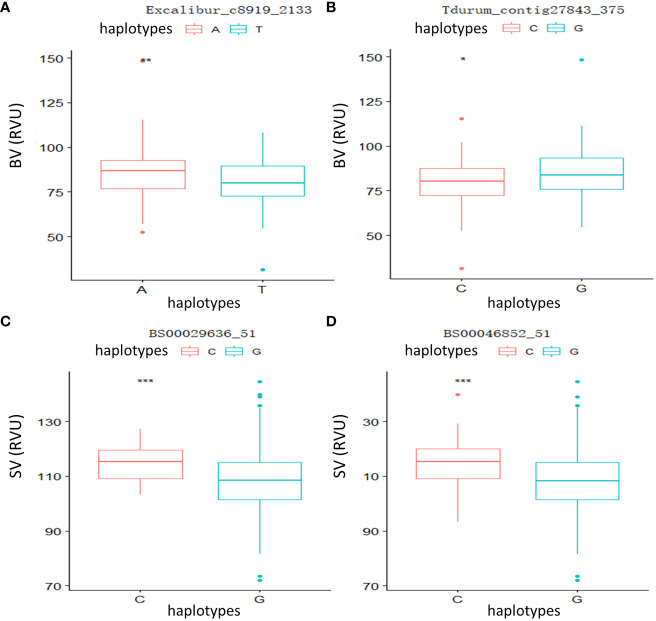
Phenotypic differences between different haplotypes at four significant loci. **(A)** Breakdown viscosity (BV): Excalibur_c8919_2133. **(B)** BV: Tdurum_contig27843_375. **(C)** Setback viscosity (SV): BS00029636_51. **(D)** SV: BS00046852_51. *p < 0.05, **p < 0.01, ***p < 0.001.

To further verify the relationship between phenotypic variation and significant loci, two QTLs linked to Tdurum_contig27843_375 and Excalibur_c8919_2133 controlling BV, as well as two major QTLs linked to BS00029636_51 and BS00046852_51 controlling SV, were selected for independent *t*-tests. The results (**Figure 3**) showed that significant and highly significant differences were observed in the parameters of the different genotypes, indicating that these candidate loci had significant effects on phenotype.

### 3.5 Identification and functional analysis of candidate genes

**Figure 4:**
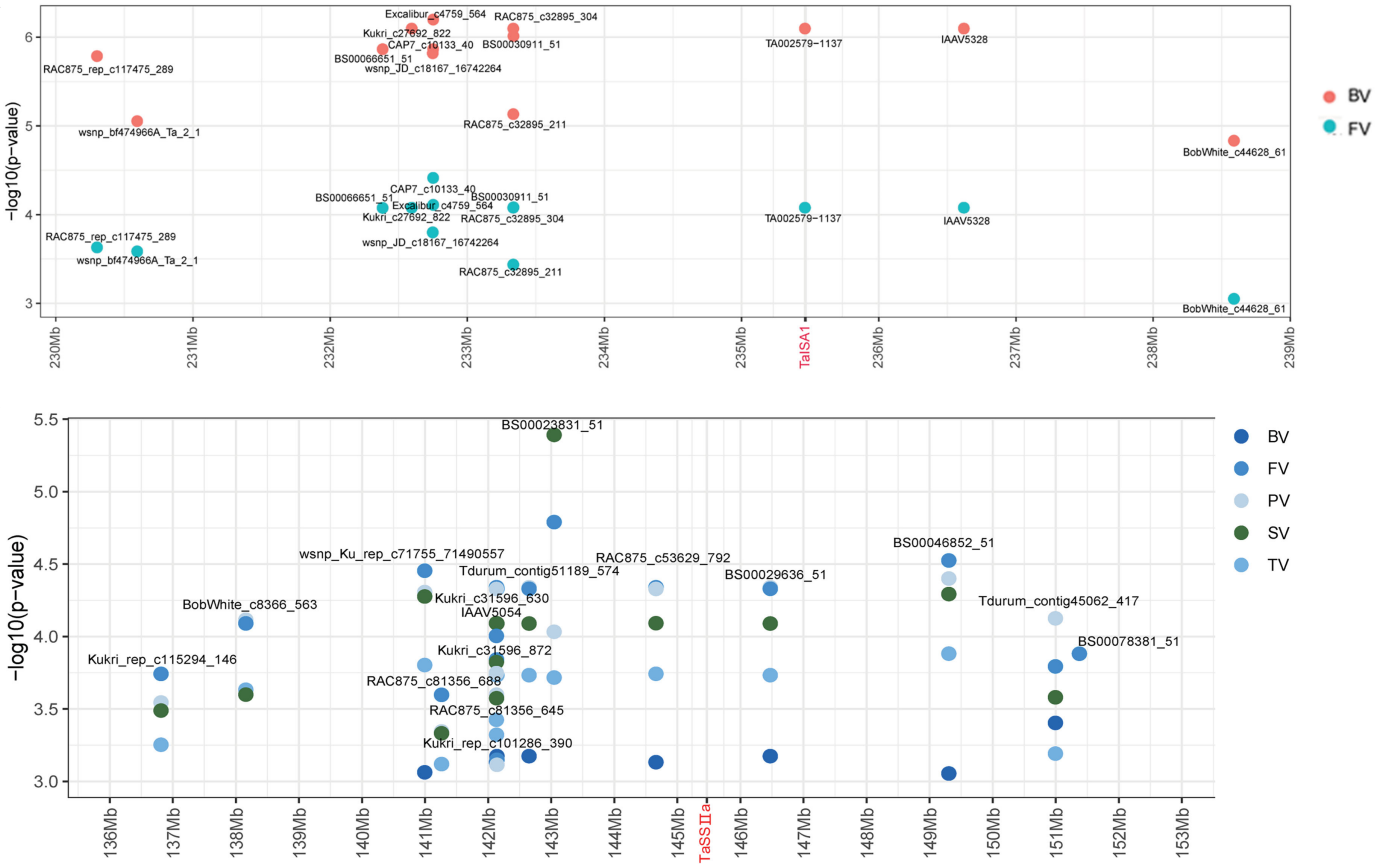
Identification of the candidate genes of *qBV7A.1*/*qPV7A* and *qBV7A.2.* **(A)** *qBV7A.1*/*qPV7A*. **(B)** *qBV7A.2*.

In this study, the 4.0-Mb sequences flanking the significant SNPs in each stable QTL associated with the parameters were regarded as potential candidate gene regions. Some putative candidate genes were identified by screening the annotated genes in the “Chinese Spring” reference genome database (IWGSC RefSeq v2.0) (**Supplementary Table S5**). For example, TA002579–1137 on Chr7A (235.46 Mb) was associated with BV and PV in two environments and overlapped with *TaISA1–7A* (*TraesCS7A02G251400*), which could be a candidate gene of the stable QTL *qBV7A.1*/*qPV7A* (**Figure 4A**).

Three candidate genes related to starch synthesis were screened for phenotyping analysis. The SNP Kukri_c47966_343 on Chr2B (382.09 Mb) was significantly associated with PV and was close to *TaSBEⅡa-2B* (*TraesCS2B02G309500*), while Tdurun_contig75336_402 on Chr2A (509.4 Mb) was significantly associated with PT and was close to *TaSBEⅡa-2A* (*TraesCS2A02G293400*). Kukri_C33620_129 on Chr7A (717.26 Mb) was associated with six RVA parameters in 2015-YL and was close to *TaSBEⅠ-7A* (*TraesCS7A02G549100*), while BS00066456_51 on Chr7B (735.29 Mb) was associated with BV and PTT in multiple environments and was close to *TaSBEⅠ-7B* (*TraesCS7B02G801300*). BS00030911_51 on Chr7A (233.34 Mb) and BS00029636_51 on Chr7A (146.47 Mb) were associated with BV, FV, TV, SV, and PV in multiple environments and were flanked on two sides of *TaSSⅡ-7A* (*TraesCS7A02G189000*), which could be candidate gene for the stable QTL *qBV7A.2* (**Figure 4B**).

**Figure 5:**
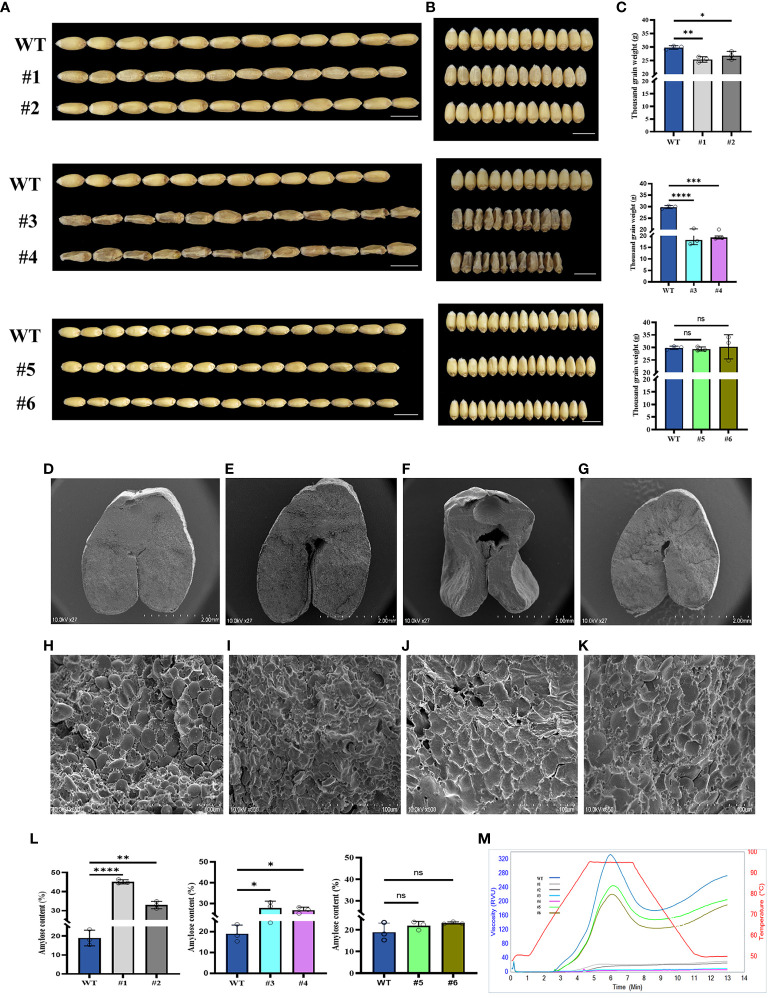
Grain phenotype and agronomic performance of grains at the maturity stage. **(A)** Grain length. **(B)** Grain width. **(C)** Thousand grain weight. **(D–G)** Transverse sections of the wild type (WT), #1, #3, and #5. *Scale bar*, 2 mm. **(H–K)** Starch granule of the WT, #1, #3, and #5 under SEM. *Scale bar*, 100 µm. **(L)** Amylose content. Values shown are the mean ± SD. **(M)** Rapid Visco Analyzer (RVA) viscograms of whole wheat flour. *, *p* < 0.05, **, *p* < 0.01, ***, *p* < 0.001, ****, *p*<0.0001. ns, no significance. #1 and #2 [knockout (KO) lines for *TaSBEⅡa*], #3 and #4 (KO lines for *TaSSⅡa*), and #5 and #6 (KO lines for *TaSBEⅠ*).

In order to analyze the phenotypes of three candidate genes, we obtained the homozygous KO lines (**Supplementary Figure S5**), #1 and #2 (*TaSBEⅡa*), #3 and #4 (*TaSSⅡa*), and #5 and #6 (*TaSBEⅠ*), and analyzed their phenotypes and RVA parameters. The results showed that the grain lengths of these KO lines were more or less similar to those of the wild type (WT) (**Figure 5A**), while the grain widths and the thousand grain weights of the *TaSBEⅡa* and *Ta*SS*IIa* KO lines were significantly decreased compared with those of the *TaSBEⅠ* KO lines and the control (**Figures 5B, C**). These results indicate that these three genes positively affected the grain size and weight in bread wheat and that *TaSBE I* had a weaker role than *TaSBEⅡa* and *TaSSⅡa*. To further observe the internal structure of the grains, the cross-sections of mature grains were analyzed with SEM, which showed that the size of endosperm cells and the A-type starch granules in the KO lines were smaller than those of the WT (**Figures 5D–K**). These traits of the KO grains suggest that the storage starch had been affected. To verify this hypothesis, we examined the starch content and composition and found that the *TaSBEⅡa* and *TaSSIIa* KO lines had significantly higher amylose content than the WT, while the *TaSBEⅠ* KO lines showed an upward trend (**Figure 5L**). Compared with the WT, the *TaSBEⅡa* KO lines had flat RVA curves, while the *TaSSIIa* lines failed to paste under the temperature conditions used for the RVA. The *TaSBEⅠ* KO lines had normal RVA curves, but their values for the RVA parameters were smaller than those of the WT (**Figure 5M**), suggesting that the *TaSBEⅠ* gene had a relatively minimal role in starch synthesis.

## 4 Discussion

### 4.1 The significantly associated loci of the RVA parameters in wheat

The gelatinization and retrogradation characteristics of wheat starch were simultaneously affected by genes and environmental factors. The significantly associated loci detected in this study were mainly located on chromosomes 2A, 2B, 2D, 6A, 6B, and 7A, consistent with previous mapping findings (Batey et al., 2002; Yu and Tian, 2012; Jin et al., 2016). The loci controlling PV were mainly located on chromosomes 1B, 2B, 2D, 4A, 5A, 6A, and 7A, which is consistent with the GWAS results of Jin et al. (2016) and the mapping results of Batey et al. (2002). A total of 144 significant loci controlling PT, mainly located on chromosomes 1B, 2A, 2B, and 4A, were also consistent with a previous mapping reported (Yu and Tian, 2012). The significantly associated loci controlling TV were mainly located on chromosomes 1B, 1A, 2A, and 6B, consistent with previous mapping results (Wu et al., 2008). Similarly, the loci significantly related to BV and SB were detected on chromosomes 2B, 2D, 6A, 6B, and 7A, which is consistent with previous QTL results (Chen, 2015). Moreover, loci related to FV were located between BS00086046_51 and BS00087178_51.

Some significant new loci/SNPs were also found, including Kukri_c33620_129, BS00110297_51, and Ku_c40588_1376, which were associated with PV; Kukri_c33620_129 and BS00092541_51, associated with TV; Kukri_c33620_129 and BS00028092_51, associated with SV; and Tdurum_contig27843_375 and Excalibur_c49801_345, which were associated with PT. These new significant loci/SNPs will be helpful for gene cloning and further molecular breeding in wheat.

### 4.2 Functional and genetic diversity of the genes related to starch synthesis in wheat and other crops

There is a significant correlation between the amylose content (AC) and the noodle quality, such as adhesion, chewiness, and retrogradation (Toyokawa et al., 1989; Li et al., 1999; Zhang et al., 2012a; Zhang et al., 2012b; Blažek and Copeland, 2008). The genes related to SS (Wx, SSI, SSII, and SSIII) and SBE (SBEI and SBEII), along with their multiple alleles, could alter the composition and structure of starch to varying degrees, thereby impacting both the RVA parameters and the starch properties.

Soluble SS catalyzed the elongation of linear chains and were considered as the main contributors in amylopectin synthesis and minor contributors in amylose synthesis. A structural gene locus controlling soluble starch aggregation was located on the chromosome of wheat homologous group 7 (Li et al., 1999). The wheat lines lacking SSII-A, SSII-B, and SSII-D had a significantly increased amylose content (30.8%–37.4%), which correlated with the rise in the proportion of side chains with a branching degree increase of 6–10, resulting in abnormal starch granule crystallization (Yamamori et al., 2000).

The grains of the *SSⅡa* mutant lines in this study were apparently damaged or somewhat shrunken. The same appearance had been previously observed in wheat and rice (Hogg et al., 2013; Jr. et al., 2020). It was observed that the A- and B-type starch granules were deformed and altered with the loss of the *SSⅡa* gene. Furthermore, the B-type granules were completely lost, as found in other studies (Yamamori et al., 2000; Morell et al., 2003; Kosar-Hashemi et al., 2007). Remarkably, the downregulation of the *ssIIa* gene showed the same RVA profile as that in previous studies (Jane et al., 1999; Shimbata et al., 2012). The extracted starch failed to paste at the temperature profile for the *SSⅡa* mutant lines with RVA. The downregulation of the expression of *SSⅡa* in wheat produced a high amylose content, as occurred in barley (Morell et al., 2003). The AC doubled in maize, from 20% to 40%, by mutating the *SSⅡa* gene, while it increased to 34% in wheat (Hanashiro et al., 2004; Zhang et al., 2004).

Modifying the expression of the wheat *SBEⅡa*, an SBE, can alter the starch content and composition and the RVA parameters. For instance, the knockdown lines 4–4-2 and 4–4-19 had weaker SBE activities (0.43 and 0.47 U g^−1^ min^−1^, respectively) than the WT (0.57 U g^−1^ min^−1^). Similarly, these knockdown lines had higher amylose contents and higher PV, TV, FV, and PT values compared with the WT (Li et al., 2014).

The downregulation of the *SBEⅡa* gene resulted in significant changes in the granule morphology, starch composition, and functional properties. The A-type granules were distorted and had an irregular shape, while the B-type granules no longer had the normal spherical shape. The results of our study were in agreement with those of Regina et al. (2006), who achieved comparable outcomes with RNA interference (RNAi). No significant impacts on the granule morphology and phenotypic changes were observed due to the loss of the *SBEⅠ* isoforms, as reported in other studies (Schwall et al., 2000; Regina et al., 2004; Sun et al., 2017). The RVA profile for the suppression of the *SBEⅡa* gene in the mutant lines aligned with those obtained for high amylose starch in corn (Juhász and Salgó, 2008), wheat (Yamamori et al., 2006), and barley (You and Kim, 2005). No significant difference in the thousand grain weight was observed for the *SBEⅡa* and *SBEⅠ* mutant lines, as previously documented (Schwall et al., 2000; Regina et al., 2004; Sestili et al., 2010; Sun et al., 2017). Increased amylose contents in the *SBEⅡa* mutant lines were detected in this study, as documented by downregulating the *SBEⅡa* gene using TILLING (targeting-induced local lesions in genomes) and RNAi, with 55% and 88.5% of amylose, respectively (Slade et al., 2012; Regina et al., 2015). There were no significant differences in the amylose content and starch structure found in the *SBEⅠ* mutants, as in previous studies in wheat, potato, and maize (Safford et al., 1998; Blauth et al., 2002; Regina et al., 2004). Conclusively, the inhibition of SBE resulted in an increase in the amylose content, thereby improving the PV, TV, and FV of the flour paste during the gelatinization process.

## Data availability statement

The datasets presented in this study can be found in online repositories. The names of the repository/repositories and accession number(s) can be found in the article/**Supplementary Material**.

## Author contributions

RU: Writing – review & editing, Writing – original draft, Data curation, Investigation. MY: Writing – original draft, Writing – review & editing, Data curation, Investigation. SL: Writing – review & editing, Data curation, Investigation. YI: Writing – review & editing, Data curation, Investigation. ZW: Writing – review & editing, Data curation, Investigation. XW: Writing – review & editing, Data curation, Investigation. JY: Writing – review & editing, Data curation, Formal analysis, Investigation, Software. BL: Writing – review & editing, Validation. ZN: Writing – review & editing, Methodology, Validation. RL: Writing – review & editing, Conceptualization, Resources, Supervision.

## Supplementary material

Supplementary Figure 1l The sub-genomic and Chromosomal distribution of SNPs used for the GWAS. **(A)**, the numbers before and after filter; **(B)**, the number distributed on each chromosome after filter.

Supplementary Figure 2 The LD decay patterns of A, B, D subgenomes and whole genome.

Supplementary Figure 3 The distribution of seven RVA parameters under 2018-LF. **(A)**, PV; **(B)**, BV; **(C)**, TV; **(D)**, PT; **(E)**, FV; **(F)**, SV; **(G)**, PTT; Y axis, frequency; X axis, viscosity.

Supplementary Figure 4 Manhattan and Quantile-Quantile (Q-Q) plots in 2015-YL. **(A)** PV; **(B)** PT; **(C)** PTT; **(D)** TV; **(E)** SV;**(F)** BV; **(G)** FV.

Supplementary Figure 5 The edited sites of the mutant lines. **(A–C)** are the Sanger sequencing results of #1 & #2 mutant lines of *TaSBEIIa* gene, #3 & #4 mutant lines of *TaSSIIa* gene and #5 & #6 mutant lines of *TaSBEI* gene, respectively, compared with wild type (WT). The blue sequences are gRNA1 and gRNA2 conserved among each gene. The red letters and dashes are base insertions and deletions respectively. The number of bases inserted ‘+’, and deleted ‘-’ are shown beside each sequence.

Supplementary Table 1 The natural population of 214 wheat cultivars/lines.

Supplementary Table 2 The primers with three sgRNAs sequences used for CRISPR/Cas9 vectors.

Supplementary Table 3 Twenty materials from the natural population with extreme phenotypic values.

Supplementary Table 4 The SNPs associated with 7 RVA parameters.

Supplementary Table 5 The SNPs of multiple RNA parameters.
