# Supplementary figures and images for: Genome-wide association study identifies loci and candidate genes for RVA parameters in wheat (Triticum aestivum L.)
Source: Front Plant Sci. 2024 Jul 22;15:1421924. doi: 10.3389/fpls.2024.1421924 (PMC11298398; doi:10.3389/fpls.2024.1421924)

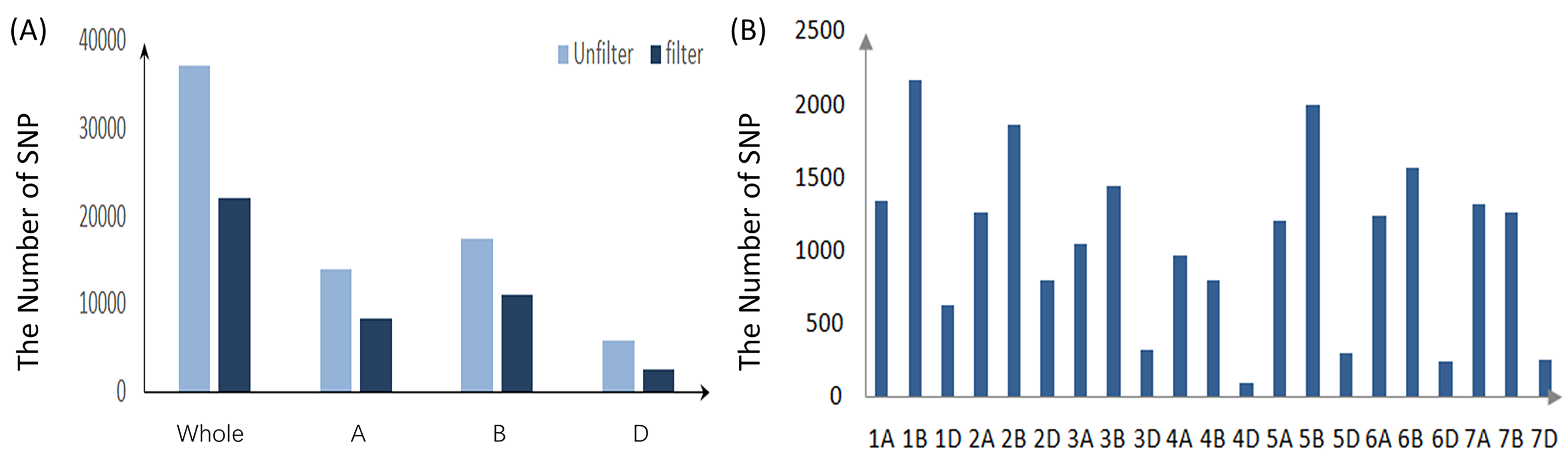

Supplement: Supplementary Figure 1l — The sub-genomic and Chromosomal distribution of SNPs used for the GWAS. (A), the numbers before and after filter; (B), the number distributed on each chromosome after filter. [file Image_1.tif]

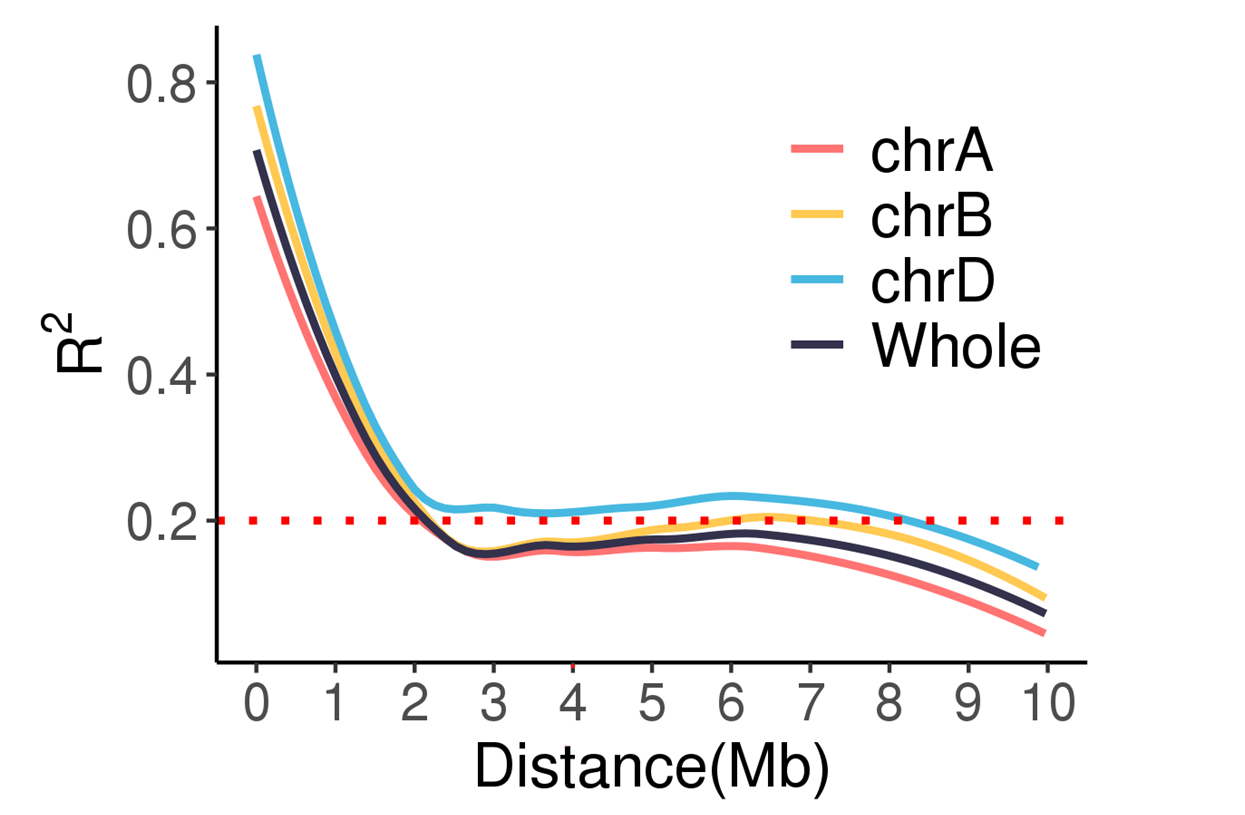

Supplement: Supplementary Figure 2 — The LD decay patterns of A, B, D subgenomes and whole genome. [file Image_2.tif]

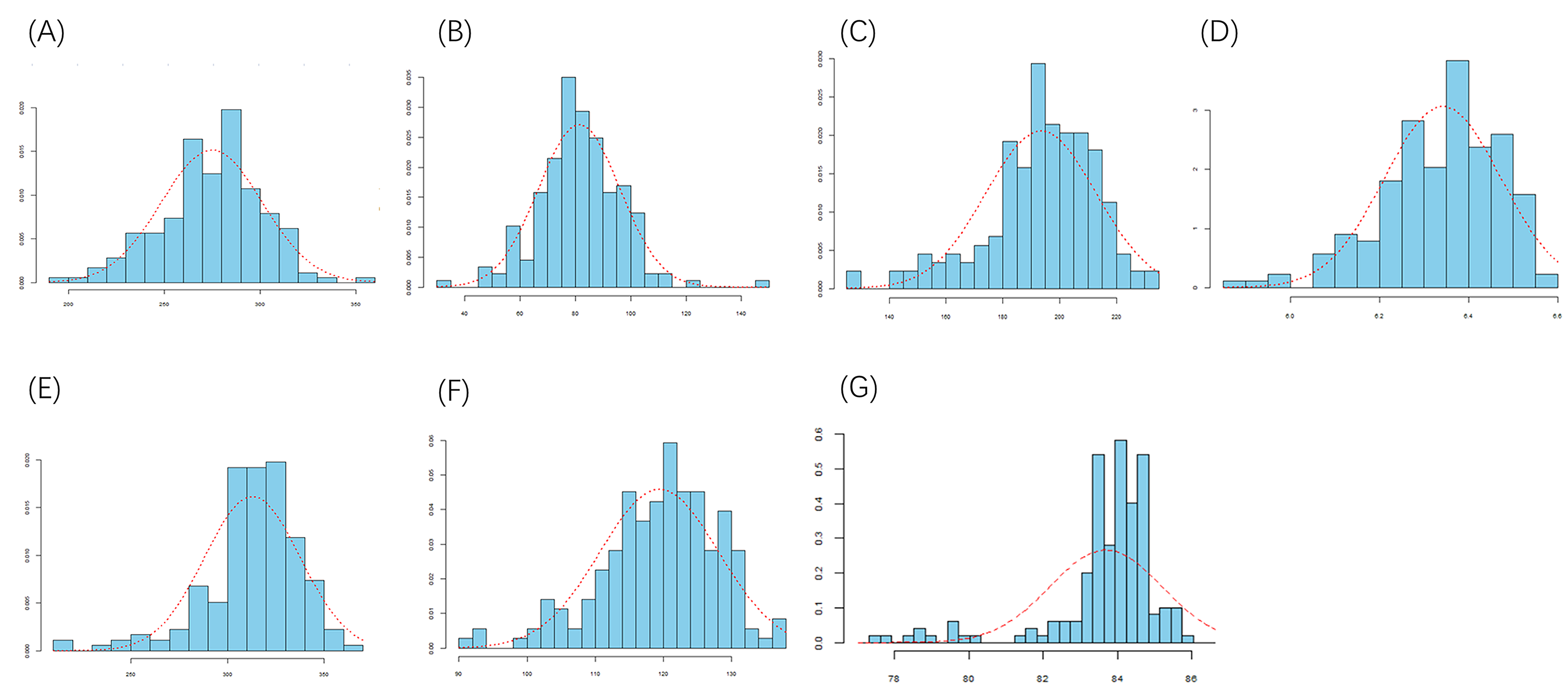

Supplement: Supplementary Figure 3 — The distribution of seven RVA parameters under 2018-LF. (A), PV; (B), BV; (C), TV; (D), PT; (E), FV; (F), SV; (G), PTT; Y axis, frequency; X axis, viscosity. [file Image_3.tif]

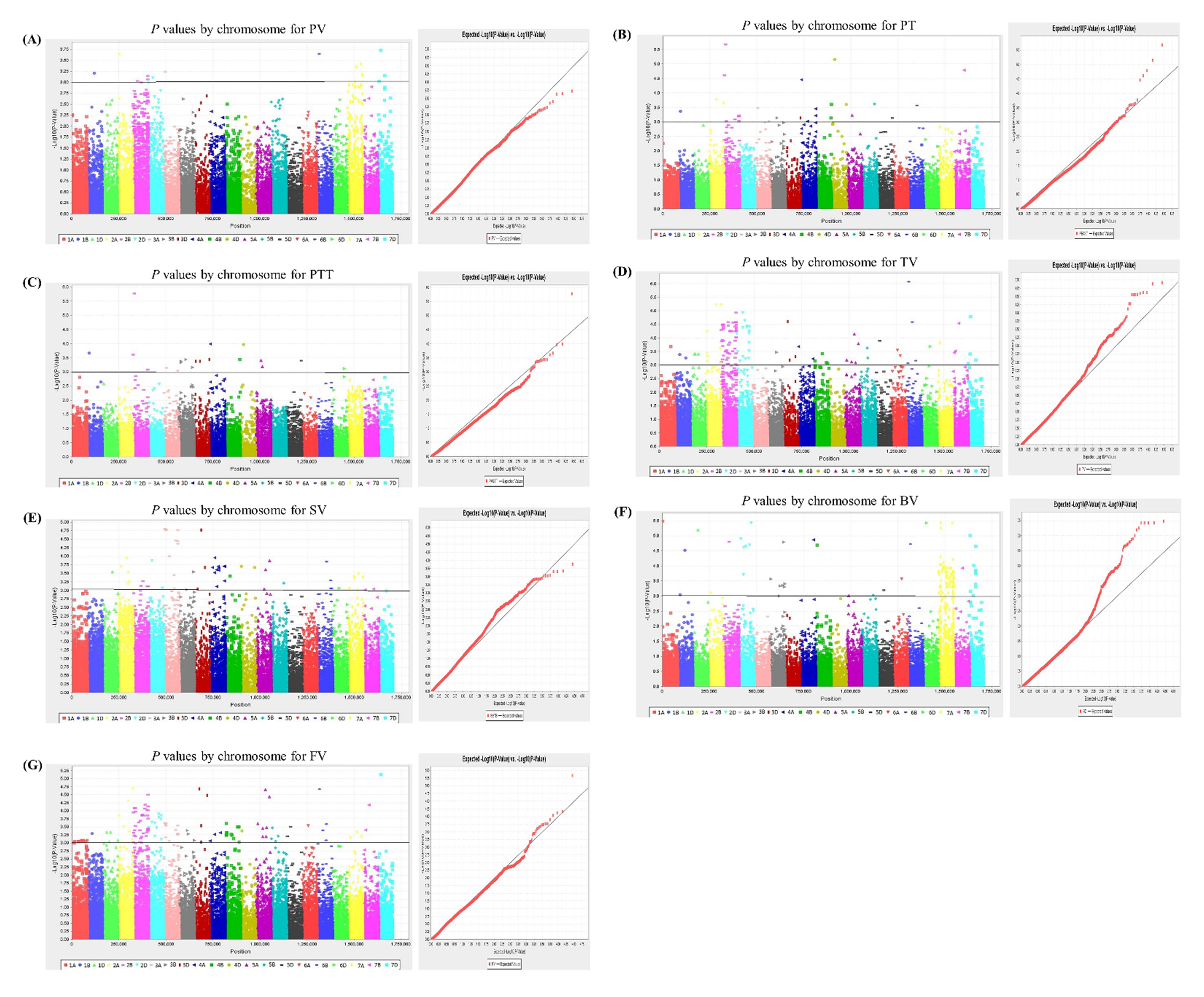

Supplement: Supplementary Figure 4 — Manhattan and Quantile-Quantile (Q-Q) plots in 2015-YL. (A) PV; (B) PT; (C) PTT; (D) TV; (E) SV;(F) BV; (G) FV. [file Image_4.tif]

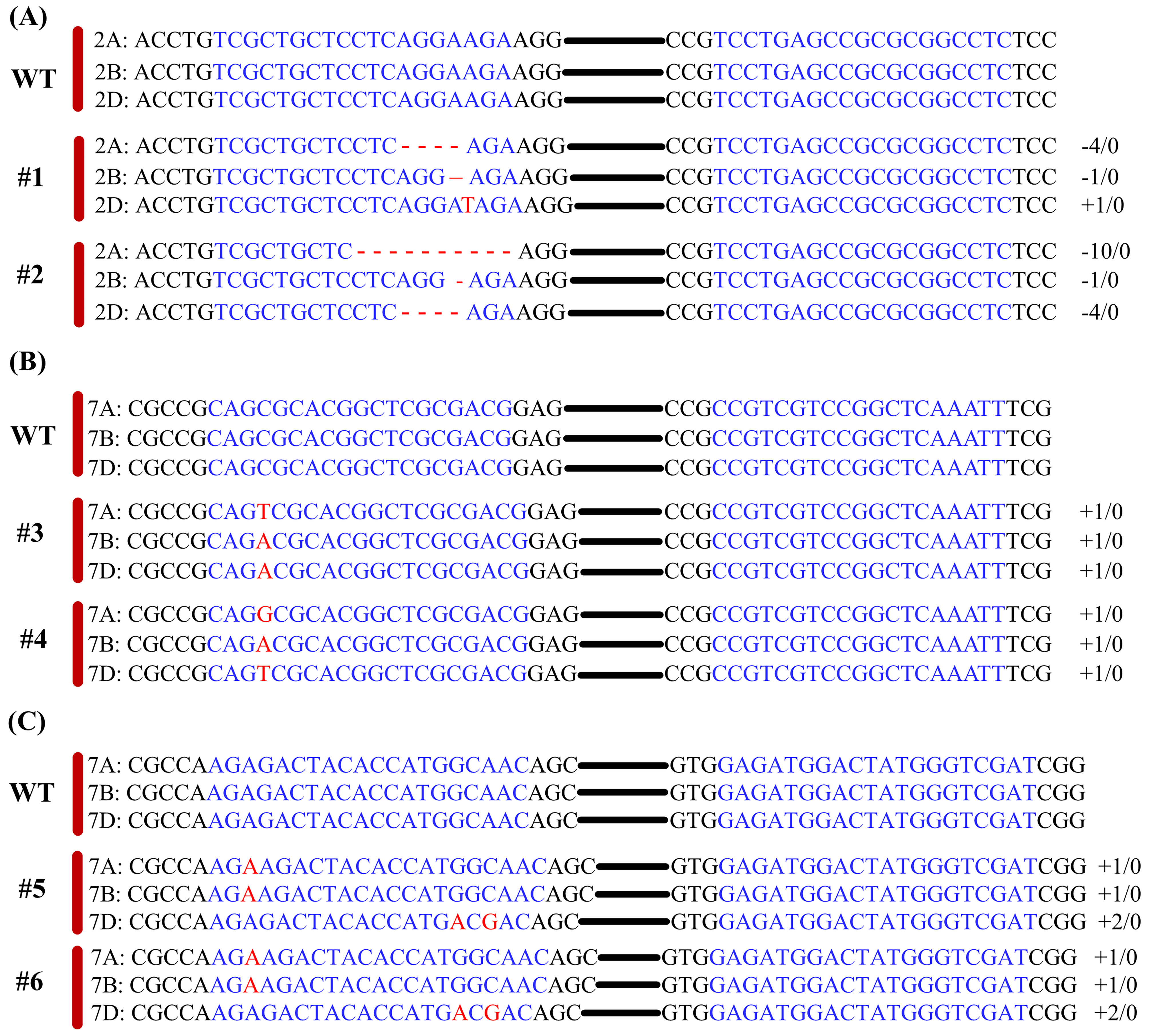

Supplement: Supplementary Figure 5 — The edited sites of the mutant lines. (A–C) are the Sanger sequencing results of #1 & #2 mutant lines of TaSBEIIa gene, #3 & #4 mutant lines of TaSSIIa gene and #5 & #6 mutant lines of TaSBEI gene, respectively, compared with wild type (WT). The blue sequences are gRNA1 and gRNA2 conserved among each gene. The red letters and dashes are base insertions and deletions respectively. The number of bases inserted ‘+’, and deleted ‘-’ are shown beside each sequence. [file Image_5.tif]
